# Overgrowth and strain investigation of (11–20) non-polar GaN on patterned templates on sapphire

**DOI:** 10.1038/s41598-018-28328-7

**Published:** 2018-07-02

**Authors:** L. Jiu, Y. Gong, T. Wang

**Affiliations:** Department of Electronic and Electrical Engineering, University of Sheffield, Mappin Street, Sheffield, S1 3JD United Kingdom

## Abstract

Non-polar (11–20) GaN with significantly improved crystal quality has been achieved by means of overgrowth on regularly arrayed micro-rod templates on sapphire in comparison with standard non-polar GaN grown without any patterning processes on sapphire. Our overgrown GaN shows massively reduced linewidth of X-ray rocking curves with typical values of 270 arcsec along the [0001] direction and 380 arcsec along the [1–100] direction, which are among the best reports. Detailed X-ray measurements have been performed in order to investigate strain relaxation and in-plane strain distribution. The study has been compared with the standard non-polar GaN grown without any patterning processes and an extra non-polar GaN sample overgrown on a standard stripe-patterned template. The standard non-polar GaN grown without involving any patterning processes typically exhibits highly anisotropic in-plane strain distribution, while the overgrown GaN on our regularly arrayed micro-rod templates shows a highly isotropic in-plane strain distribution. Between them is the overgrown non-polar GaN on the stripe-patterned template. The results presented demonstrate the major advantages of using our regularly arrayed micro-rod templates for the overgrowth of non-polar GaN, leading to both high crystal quality and isotropic in-plane strain distribution, which is important for the further growth of any device structures.

## Introduction

There is a significantly increasing demand on III-nitride based power electronics and radio-frequency (RF) devices required for 5G mobile communications, as GaN exhibits major advantages in fabricating high power, high frequency and high temperature devices due to its intrinsically high breakdown voltage, high saturation electron velocity and excellent mechanical hardness^1,2^. So far, the development of III-nitride electronics is built on AlGaN/GaN heterostructure field transistors (HFETs) grown on *c-plane* GaN surface^3,4^. This polar orientation poses strong polarisation at the interface between AlGaN and GaN, leading to a high sheet carrier density of up to 10^13^/cm^2^ obtained without modulation doping as a result of spontaneous and piezoelectric polarisation^5,6^. On the one hand, this is the major advantage for III-nitride electronics grown on *c-plane* GaN surface. On the other hand, such polarisation generally leads to a depletion-mode transistor as a consequence, while it is ideal to obtain enhancement-mode devices in practical applications due to safety requirements. Any change in strain would also affect the electrical performance of such HFETs, for instance, SiN deposition required for surface passivation, which could potentially lead to degradation in performance or even reliability issues^7–9^. Furthermore, the sheet carrier density of a two dimensional electron gas (2DEG) formed at the interface between GaN and AlGaN sensitively depends on the polarisation, and thus cannot be simply tuned in a controllable manner as required.

Based on the well-established experience built on the growth and fabrication of AlGaAs/GaAs high electron mobility transistors (HEMTs), a simple but promising solution is to grow an AlGaN/GaN heterostructure with modulation doping along a non-polar direction, where the polarisation can be eliminated and thus the sheet carrier density of 2DEG can be tuned simply through optimising the doping level in AlGaN barrier^6^.

However, the crystal quality of current non-polar GaN grown on either sapphire or silicon is far from satisfactory. Typically, non-polar GaN grown on sapphire without any extra processes exhibits a high density of defects (a dislocation density of above 10^10^/cm^2^ and a stacking fault density of above 10^6^/cm)^10,11^. Therefore, it is crucial to develop a new method for the growth of non-polar GaN on sapphire or silicon substrates, the industry-compatible substrates.

Previously, we developed a cost-effective approach for the overgrowth of (11–22) semi-polar GaN on *m-plane* sapphire by using regularly arrayed micro-rod templates, leading to substantial improvement in crystal quality. By using the semi-polar GaN templates, we have achieved high performance light emitting diodes (LEDs) with longer emission wavelengths such as green and amber^12–15^. We further extend this approach to the overgrowth of non-polar (11–20) GaN on *r-plane* sapphire, aiming to significantly improve the overall performance of non-polar GaN including crystal quality and strain situation that is a typical issue for GaN grown on large lattice-mismatched substrates. The strain issues of non-polar GaN grown on any foreign substrates such as sapphire or silicon are much more complicated than those of its *c-plane* counterpart. Non-polar GaN grown on large lattice-mismatched substrates intrinsically exhibits a strongly anisotropic in-plane strain distribution due to the absence of six-fold-symmetry. In return this anisotropic in-plane strain distribution leads to anisotropy in electrical performance and also challenges in device fabrication^16^. Furthermore, it is much more complicated to precisely determine the lattice parameters of non-polar GaN than its *c-plane* counterpart^17–21^. The strain of any overgrown non-polar GaN also depends on the patterned templates employed for conducting the overgrowth, as any residual voids left as a consequence of overgrowth processes lead to strain relaxation. This offers a great opportunity to redistribute strain or even eliminate strain.

In this work, we present a detailed study of strain relaxation of our (11–20) non-polar GaN with high crystal quality which has been achieved by means of overgrowth on our regularly arrayed micro-rod templates. Detailed X-ray measurements along symmetric and skew-symmetric directions have been employed for the study. For comparison we have also performed overgrowth of non-polar GaN on a standard stripe-patterned template which is typically used for GaN overgrowth and then have studied its strain relaxation as well. Our results indicate that the overgrown GaN on our regularly arrayed micro-rod templates exhibits a highly isotropic in-plane strain distribution, while standard non-polar GaN grown without any patterning processes on sapphire exhibits a strongly anisotropic in-plane strain distribution. Between them is the overgrown GaN on the stripe-patterned template in terms of in-plane strain distribution. We have also presented the evolution of the surface morphology and the crystal quality of the overgrown non-polar GaN on our regularly arrayed micro-rod templates. Of course, the results presented have also confirmed significantly improved crystal quality of the (11–20) non-polar GaN overgrown on our regularly arrayed micro-rod templates.

A single (11–20) non-polar GaN layer with a thickness of 1 µm is initially grown on *r-plane* sapphire using our high temperature AlN buffer approach by a low-pressure metalorganic vapour phase epitaxial (MOVPE) technique^22^, labelled as “as-grown” template. Subsequently, the as-grown template is fabricated into a regularly arrayed micro-rod template by means of a standard photolithography technique and subsequent dry-etching processes.

**Figure 1:**
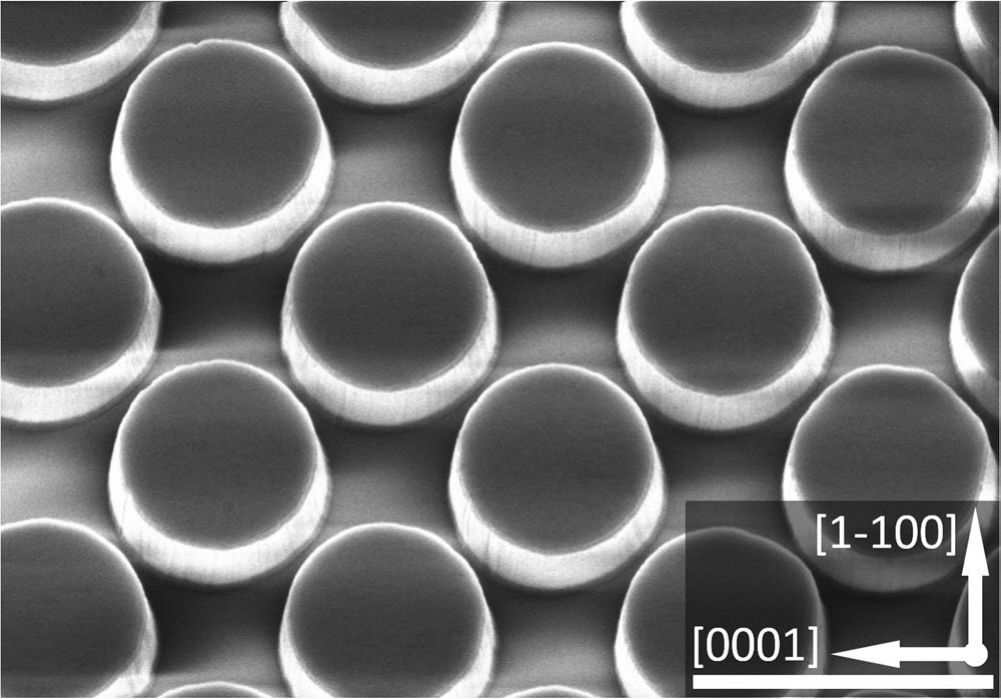
Plane-view SEM image of our regularly arrayed micro-rod array template. Scale bar: 4 μm.

Figure 1 shows a typical scanning electron microscopy (SEM) image of our regularly micro-rod array template which exhibits a chess-board configuration. The diameter of each micro-rod is 2.5 µm. It is well-known that the overgrowth of semi- or non- polar GaN along the [0001] direction, i.e., *c* direction, leads to being defect-free, while the overgrowth along the opposite direction (i.e., *-c* direction) allows defects to effectively penetrate to any overlying layers^13–15^. Therefore, it is essential to ensure that the growth along the *c* direction is dominant and that the growth along the *-c* direction can be effectively suppressed. This can be achieved by optimising growth conditions such as increasing growth temperatures. Our unique pattern configuration is expected to not only enhance improvement in crystal quality, but also help compensate the intrinsically anisotropic growth rate of non-polar GaN, leading to quick coalescence and then a smooth surface.

For the fabrication of a standard stripe-patterned template^23,24^, (11–20) non-polar GaN stripes with a width of 1.5 μm and a spacing of 1.5 μm between stripes are formed. These parallel stripes formed orientate along the [1–100] direction, which is perpendicular to the *c* direction of (11–20) non-polar GaN. Similarly, the etching is also performed down to the sapphire substrate, and the formed SiO_2_ strip mask remains on top of each GaN stripe.

**Figure 2:**
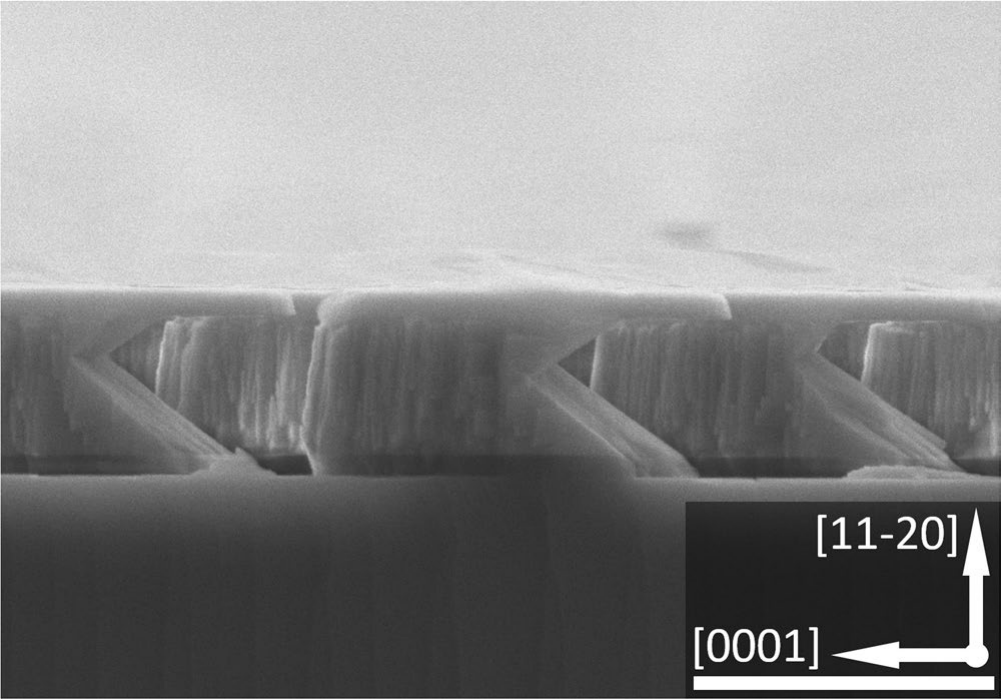
Cross-sectional SEM image of our micro-rod with a “mushroom” configuration. Scale bar: 2 μm.

In each case, prior to any overgrowth the patterned GaN templates further undergo photo-enhanced chemical (PEC) etching using 10% potassium hydroxide (KOH) in order to partially remove GaN from the [000–1] direction which is nitrogen-face, while the Ga-face GaN from the [0001] direction, i.e., *c* direction, remains almost un-etched. The PEC etching does not etching SiO_2_ masks. As a result, the GaN micro-rods are finally formed into a mushroom configuration as shown in Fig. 2. By using the micro-rod arrays with a mushroom configuration, the overgrowth along the [000-1] direction can be effectively suppressed, thus reducing the penetration of defects significantly. Therefore, it is expected that this mushroom configuration leads to further improvement in crystal quality. Finally, the regularly arrayed micro-rod template is reloaded into the MOVPE chamber for overgrowth. For the present study, the overgrowth has been performed on the regularly arrayed micro-rod templates under identical conditions except growth temperatures. The overgrown samples are grown at 1100 °C, 1120 °C and 1140 °C, labelled as Sample A, Sample B and Sample C, respectively. For comparison, the overgrowth on the standard stripe-patterned template has also been conducted under identical conditions at 1100 °C, denoted as Sample D.

**Figure 3:**
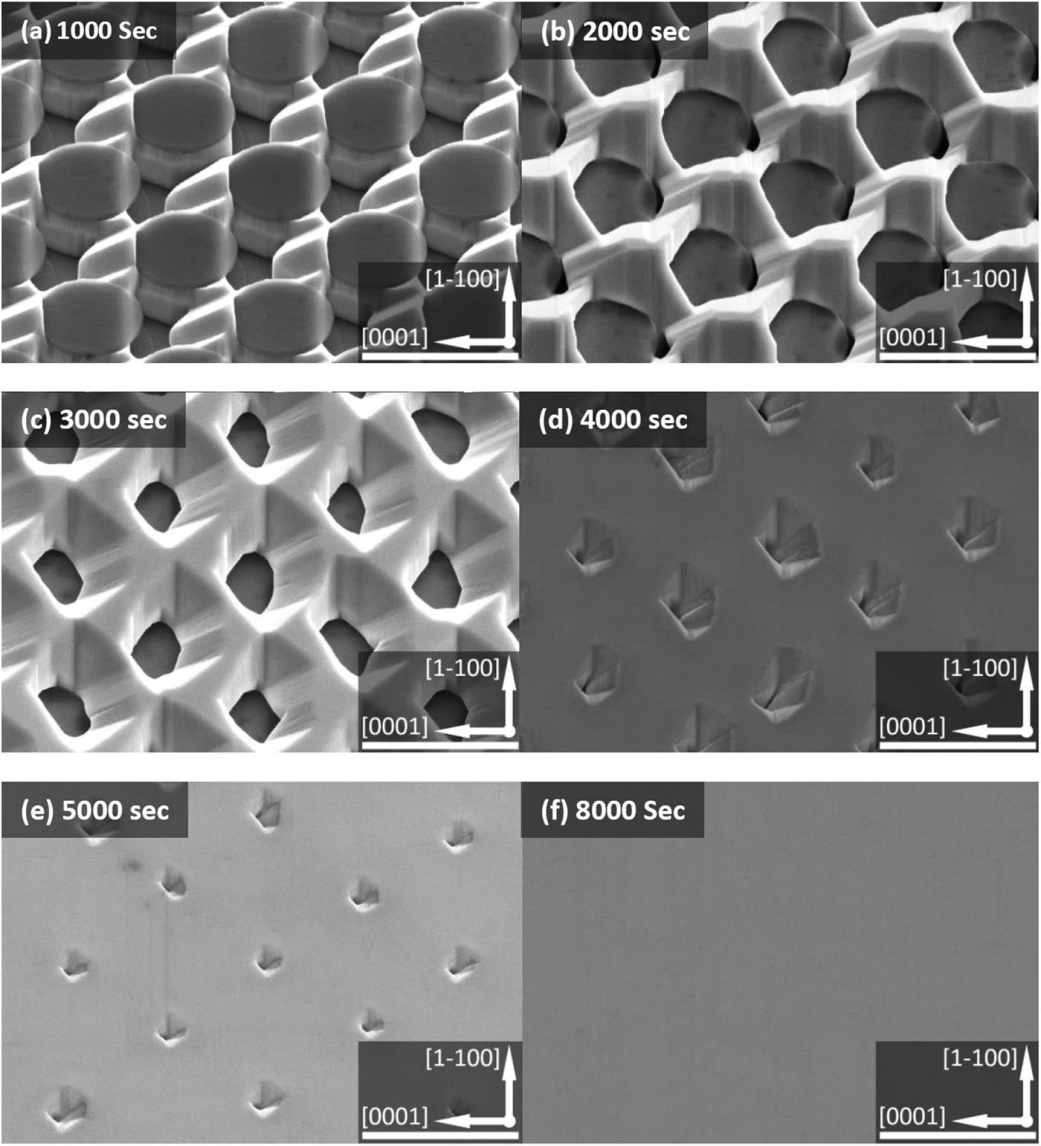
Plane-view SEM images of the nonpolar GaN overgrown on our regularly arrayed micro-rod array template as a function of growth time from 1000 to 8000 seconds. Scale bar: 4 μm.

Figure 3 shows the plane-view SEM images of the overgrown GaN on our micro-rod template as a function of overgrowth time from 1000 to 8000 seconds, demonstrating its surface evolution and its coalesce processes. Figure 3a indicates that the overgrowth initiates mainly along the [0001] direction (i.e., *c* direction) and partially along the [1–100] and [−1100] directions, while the overgrowth along the [000–1] direction (i.e., *-c* direction) has been effectively suppressed. During the first 1000 seconds, the overgrown GaN exhibits a truncated triangular prism around each micro-rod. These truncated triangular prisms have partly merged with each other, indicating that the first coalescence starts to occur before 1000 seconds. The first coalescence process completes before 2000 seconds as shown in Fig. 3b, where the gap between the micro-rods have been completely filled due to lateral overgrowth, and the vertical overgrowth has become dominant. The second coalescence happens once the lateral growth starts to extend to above the SiO_2_ masks. During this process, multiple semi-polar facets are generated as shown in Fig. 3c. Figures 3d–f show that full coalescence can be gradually completed after 5000 seconds, finally forming a smooth surface.

High resolution X-ray diffraction measurements have been performed in order to evaluate crystal quality and study strain issues. Due to the anisotropic nature of non-polar GaN, it is necessary to measure the full width at half maximum (FWHM) of an XRD rocking curve as a function of azimuth angle.

**Figure 4:**
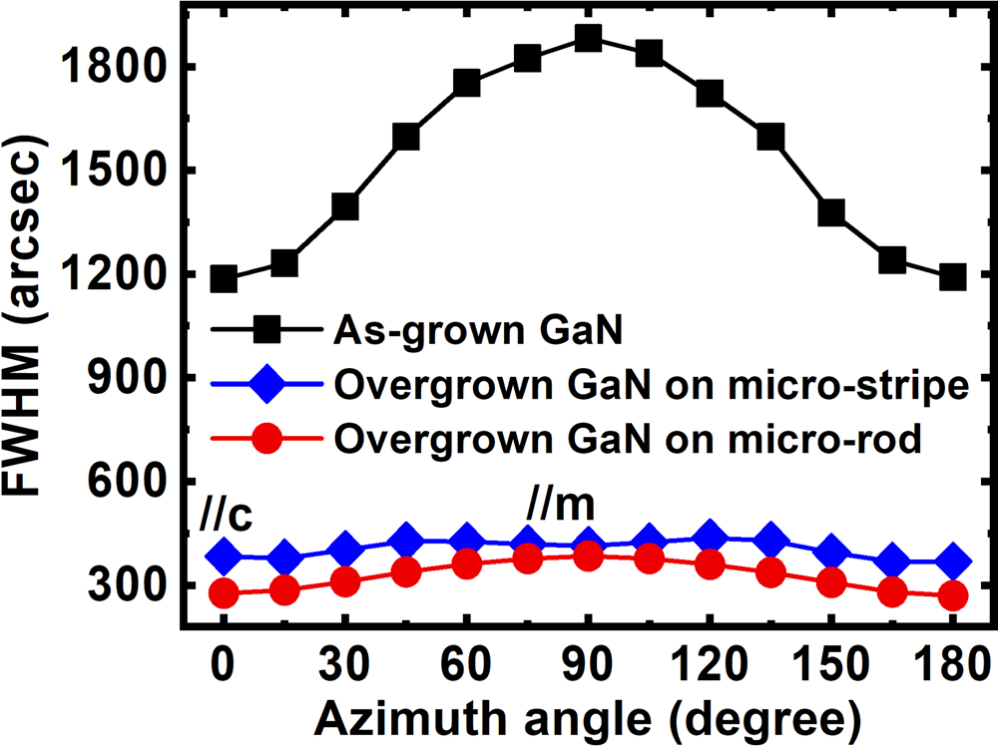
FWHM of the (11–20) on-axis XRD rocking curves as a function of azimuth angle for the as-grown sample, the overgrown GaN on our regularly arrayed micro-rod template, and the overgrown GaN on the standard stripe-pattern template.

Figure 4 shows the FWHMs of the on-axis XRD rocking curves of the two overgrown samples (one on our regularly micro-rod array template and another on the standard stripe-patterned template) as a function of azimuth angle from 0° to 180°. For comparison, Fig. 4 also includes the data of the as-grown template. For any non-polar (11–20) GaN, the smallest FWHM typically appears at the 0° azimuth angle, while the largest FWHM is normally at the 90° azimuth angle.

Figure 4 shows that the as-grown GaN template typically exhibits very large FWHMs along both the [0001] and the [1–100] directions, which are 1180 and 1880 arcsec, respectively. In remarkable contrast, the overgrown GaN on our regularly arrayed micro-rod template exhibit a massive reduction in FHWM of XRD rocking curve, which has been down to 270 arcsec along the [0001] direction and 380 arcsec along the [1–100] direction, respectively. These values are the best report for (11–20) non-polar GaN on sapphire so far, demonstrating significant improvement in crystal quality. Our detailed transmission electron microscopy measurements (which will be published elsewhere) indicate that the dislocation density has been reduced from ~10^11^/cm^2^ for the as-grown template to ~6 × 10^8^/cm^2^ for our overgrown sample.

The overgrown sample with a similar thickness on the standard stripe-patterned template shows broader FWHMs of XRD rocking curves than those of the overgrown samples on our regularly arrayed micro-rod templates. They are 380 arcsec along the [0001] direction and of 420 arcsec along the [1–100] direction, respectively.

The above data demonstrate the advantage of using our regularly arrayed micro-rod template for the overgrowth of (11–20) non-polar GaN on *r-plane* sapphire.

In order to study strain distribution, X-ray reciprocal space mapping (RSM) measurements have been carried out on all the samples. The RSM have been measured on an asymmetric plane, i.e., the (11–22) plane, where the lattice constants for both the in-plane and the out-of-plane can be measured simultaneously.

**Figure 5:**
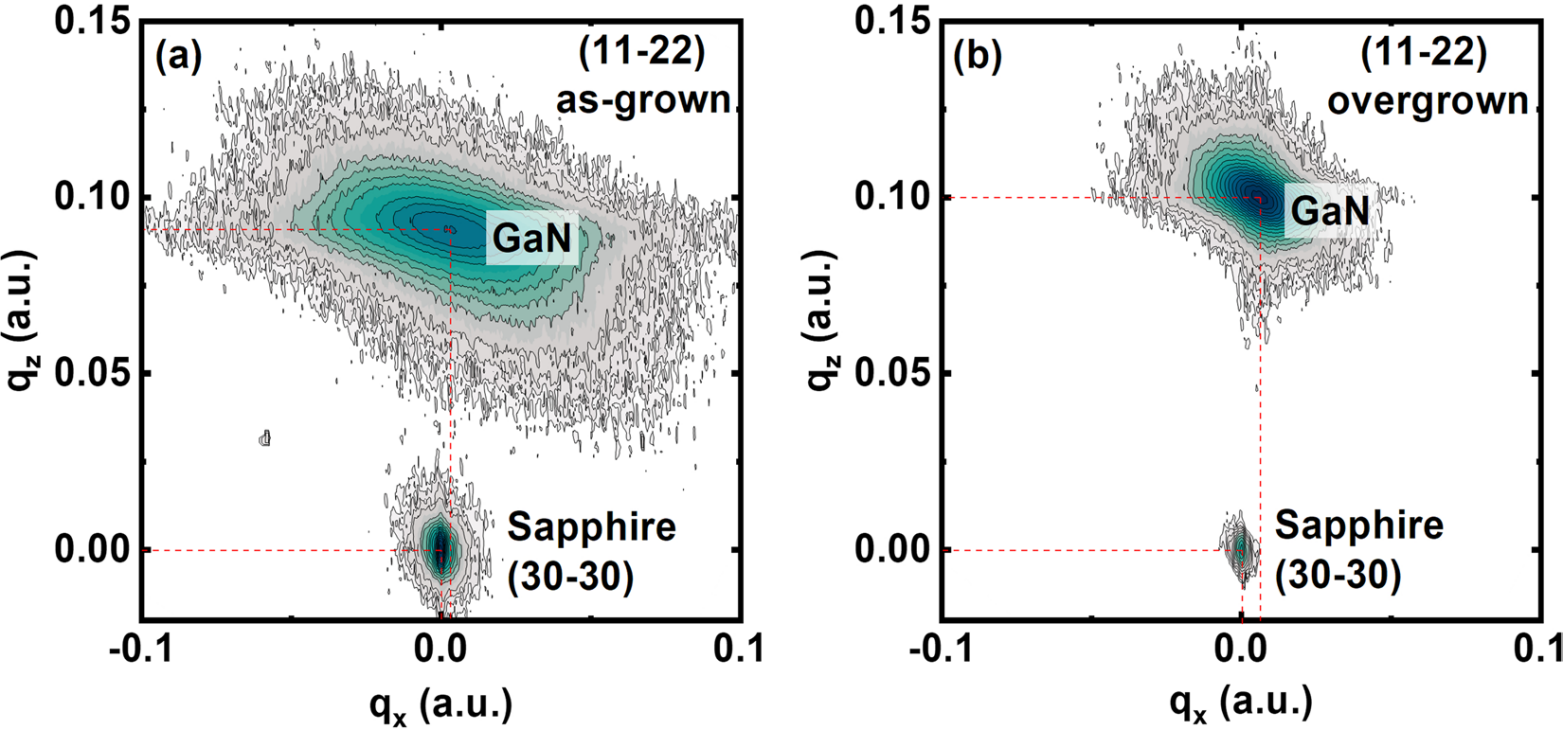
RSM of (**a**) the as-grown sample, and (**b**) the overgrown sample on our regularly arrayed micro-rod template.

Figure 5 shows the typical (11–22) RSM of the as-grown sample and the overgrown sample on our regularly arrayed micro-rod template, where the sapphire substrate is used as a reference. The sapphire substrate is 430 µm thick and is much thicker than any epitaxial layers. The coordinates of q_x_ and q_z_ represent the interplanar spacing along the in-plane [0001] direction and the out-of-plane [11–20] direction, which are proportional to 1/*c* and 1/*a*, respectively. *c* represents the in-plane lattice constant, while *a* is the lattice constant along the vertical direction.

Figure 5 indicates that the overgrown GaN exhibits smaller *c* and *a* in comparison with an unstrained case, demonstrating that the overgrown GaN suffers from compressive strain along the [0001] direction.

However, it is not good enough to only use the data obtained along the [0001] direction to investigate the anisotropy of the in-plane strain distribution of (11–20) non-polar GaN. The RSM data along the *m* direction which is perpendicular to the [0001] direction are also necessary. Unfortunately, due to the absence of the XRD peak of *r-plane* sapphire along this particular direction, there is not a sapphire reference for this direction.

Alternatively, the in-plane strain of (11–20) GaN can be studied by means of performing XRD measurements in a *ω*/*2θ* mode, where multiple symmetric and skew-symmetric reflections can be obtained. Unlike *c-plane* GaN, due to reduced symmetry (11–20) GaN exhibits anisotropic biaxial strain which results in an orthorhombic distortion. This leads to a great challenge in precisely determining lattice constants^17–21^. Therefore, an approach developed by Laskar *et al*. can be used to accurately determine the lattice constants, where they takes the distortion into account and then use least-square method for error-minimization^21^. In our case, we firstly measure the interplanar spacing (labelled as *d*_hkl_) of a number of planes of non-polar GaN, which are (11–20), (2-1-10), (11–22), (10–12), (20–20), (21–30), (21–32) planes, respectively. Equation 1 given below is then used to determine the lattice parameters in a strained state^21^:

1 $$[\tfrac{{\rm{4}}}{{\rm{3}}}({{h}}^{2}+{{k}}^{2}+{hk})]\tfrac{1}{{{a}}^{2}}-[\tfrac{{\rm{4}}}{{\rm{3}}\sqrt{{\rm{3}}}}(2{{h}}^{2}+2{{k}}^{2}+5{hk})]\tfrac{{\delta }}{{{a}}^{2}}+({l}^{2})\tfrac{1}{{{c}}^{2}}=\tfrac{1}{{{d}}_{{hkl}}^{2}}$$

where *a* and *c* are the lattice constants; *δ* is the offset of basis angle *γ;* h, l, k are the Miller indices.

Once the lattice parameters are obtained, the strain can then be calculated using a group of equations below:

2 $${{\varepsilon }}_{{\rm{xx}}}={{\varepsilon }}_{\mathrm{11}-\mathrm{20}}=\frac{{{a}}_{{s}}-{{a}}_{0}}{{{a}}_{0}}$$

3 $${{\varepsilon }}_{{\rm{zz}}}={{\varepsilon }}_{{\rm{0001}}}=\frac{{{c}}_{s}-{{c}}_{0}}{{{c}}_{0}}$$

4 $${{\varepsilon }}_{{\rm{yy}}}={\varepsilon }_{1-\mathrm{100}}=\frac{{{m}}_{s}-{{m}}_{0}}{{{m}}_{0}}$$

where ε_xx_ is the out-of-plane strain along the [11–20] direction (i.e., the growth direction), ε_zz_ and ε_yy_ are the in-plane strain along the [0001] direction (i.e., the *c* direction) and the [1–100] (i.e., the *m* direction which is perpendicular to the *c* direction), respectively. *a*_*s*_ is the lattice constant in a strained state along the vertical direction, while *c*_*s*_ *and m*_*s*_ are the in-plane lattice constants along the *c* direction and the *m* direction in a strained state, respectively. *a*_0_*, c*_0_ *and m*_0_ represent the corresponding lattice constants in a fully relaxed state.

The stress along the growth direction σ_xx_ is naturally zero. The stress along the *c* direction and the *m* direction, labelled as σ_zz_, and σ_yy,_ respectively, can be expressed by:

5 $${{\sigma }}_{{\rm{zz}}}={{C}}_{13}{{\varepsilon }}_{{xx}}+{{C}}_{13}{{\varepsilon }}_{{yy}}+{{C}}_{33}{{\varepsilon }}_{{zz}}$$

6 $${{\sigma }}_{{\rm{yy}}}={{C}}_{12}{{\varepsilon }}_{{xx}}+{{C}}_{11}{{\varepsilon }}_{{yy}}+{{C}}_{13}{{\varepsilon }}_{{zz}}$$

where *C*_ij_ are the elastic stiffness coefficients. All the parameters used for the present study are listed in Table 1^25,26^.

**Table 1:** Lattice constants and elastic stiffness coefficients of GaN in an unstrained state.

Lattice Parameters (Å)			Elastic Stiffness Constants (GPa)			
*a*_0_	*c*_0_	*m*_0_	*c*_11_	*c*_12_	*c*_13_	*c*_33_
3.1893	5.1851	2.7620	390	145	106	398

Table 2 shows all the strain related data which are obtained based on the discussion above and our detailed XRD measurements. The “+” and “−” signs in Table 2 indicate tensile and compressive strain, respectively.

**Table 2:** Lattice constants, interplanar spacing, distorted angle, strain and stress of all the (11–20) GaN samples.

Sample	*a*_*s*_ (Å)	*c*_*s*_ (Å)	*m*_*s*_ (Å)	*γ* (deg)	*ε*_11–20_ (%)	*ε*_0001_ (%)	*ε*_1–100_ (%)	σ_zz_ (GPa)	σ_yy_ (GPa)
Sample A	3.1926	5.1769	2.7579	119.87	+0.102	−0.158	−0.150	−0.6810	−0.6051
Sample B	3.1926	5.1769	2.7578	119.87	+0.103	−0.158	−0.152	−0.6813	−0.6123
Sample C	3.1928	5.1764	2.7575	119.86	+0.109	−0.169	−0.164	−0.7297	−0.6602
Sample D	3.1944	5.1810	2.7571	119.83	+0.160	−0.080	−0.178	−0.3371	−0.5472
As-grown	3.1935	5.1799	2.7540	119.79	+0.132	−0.101	−0.291	−0.5704	−1.0487

The lattice constants *a* and *c* obtained are in good agreement with the RSM measurements discussed above. Table 2 also demonstrates that all the samples generally exhibit compressive in-plane strain along both the [0001] and [1–100] direction, while the strain along the out-of-plane direction is tensile. This agrees with the previous report based on a fact that the unit cell of *r-plane* sapphire is smaller than that of *a-plane* GaN (i.e., non-polar GaN) grown on sapphire^27^.

For the as-grown template, the strain along the [1–100] direction is nearly three times higher than that along the [0001] direction, showing strong anisotropy. This anisotropic strain caused is due to the remarkable contrast of the lattice mismatch between *r-plane* sapphire and *a*-*plane* GaN along the [1–100] and [0001] directions, which are 16% and 1%, respectively. For the overgrown GaN on the stripe-patterned template, Table 2 shows that the strain along the [1–100] is almost twice as that along the [0001] direction, indicating that the in-plane strain is still highly anisotropic. This can be simply understood from the overgrowth process: the overgrowth initiates from the sidewalls of stripes, then advances along the [0001] direction only and finally leads to coalescence with the formation of voids underneath but not along the [1–100] direction due to the stripe configuration. Consequently, the overgrowth along the [0001] direction is free to proceed until coalescence, while the growth along the [1–100] direction is confined as a result of the stripes which orientate along the [1–100] direction throughout the whole overgrowth process. Therefore, strain relaxation can easily occur along the [0001] direction, but it does not effectively take place along the [1–100] direction.

Table 2 shows that all the overgrown GaN samples (i.e., Sample A, B and C) on our regularly arrayed micro-rod templates exhibit isotropic in-plane strain. This is due to our micro-rod pattern, as the overgrown GaN can laterally proceed along all the in-plane directions, although the growth rates are different. Finally, the coalescence is completed with forming voids along all the directions. Therefore, such a growth mode leads to strain relaxation along all the directions, thus forming an isotropic in-plane distribution.

**Figure 6:**
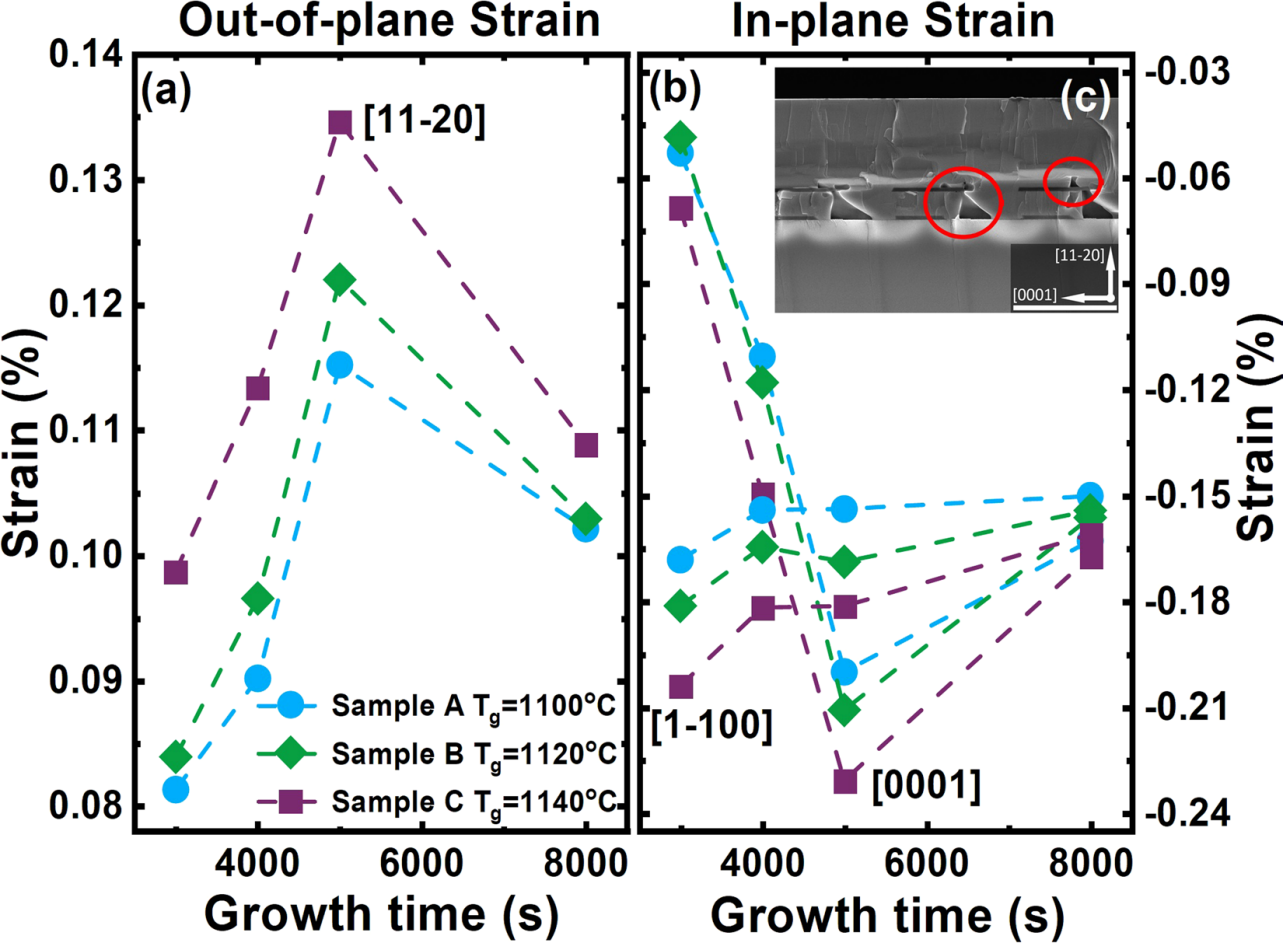
(**a**) Out-of-plane strain evolution of Sample A, B and C as a function of overgrowth time; (**b**) in-plane strain evolution of Sample A, B and C as a function of overgrowth time. Inset: Cross-sectional SEM image of a fully coalesced sample overgrown on our regularly arrayed micro-rod template. The red circles highlight two kinds of voids. Scale bar: 2 μm.

Figure 6 shows the strain of our overgrown GaN samples on the regularly arrayed micro-rod templates grown at different temperatures as a function of overgrowth time, demonstrating how the strain evolves corresponding to the overgrowth evolution as shown in Fig. 3. Figure 6 shows that the strain along all the directions generally increases with increasing growth temperature. This is consistent with a fact that thermal strain increases with increasing growth temperature.

Figure 6 also indicates that the strain increases with increasing overgrowth time of up to 5000 seconds, where the growth process evolves from initial growth from the sidewalls to almost full coalescence, confirmed from Fig. 3. Further increasing overgrowth time leads to the completion of the formation of two kinds of voids (**Inset** shows a cross-sectional SEM image, demonstrating one kind of void formed between micro-rods and another kind of void formed on top of each SiO_2_ mask). Consequently, the strain gradually reduces after 5000 seconds.

In conclusion, by means of overgrowth on regularly arrayed micro-rod templates (11–20) non-polar GaN with significantly improved crystal quality has been obtained, leading to the record low FWHM of XRD rocking curves achieved on *r-plane* sapphire. A systematic study of strain relaxation and in-plane strain distribution has been carried out by performing detailed X-ray measurements along symmetric and skew-symmetric directions. These results have been compared with the standard non-polar GaN grown on *r-plane* sapphire without involving any patterning processes and the overgrown sample on the widely used stripe-pattern template. This comparison demonstrates that the as-grown sample exhibits a highly anisotropic in-plane distribution, while the overgrown GaN on our regularly arrayed micro-rod template shows an isotropic in-plane distribution. Between them is the overgrown sample on the standard stripe-patterned template.

## Methods

### Fabrication of patterned templates

A 150 nm SiO_2_ film is firstly deposited on the as-grown template by using a standard plasma enhanced chemical vapour deposition (PECVD) technique, and then the SiO_2_ film is fabricated into regularly arrayed micro-rods with a micro-rod diameter of 2.5 μm by means of a standard photolithography technique and then dry etching processes using a reactive ion etching (RIE). The regularly arrayed SiO_2_ micro-rods are served as a second mask to finally etch the GaN layer underneath into regularly arrayed GaN micro-rods by using a standard inductively coupled etching (ICP) system. The GaN etching is performed down to the sapphire substrate. Each SiO_2_ micro mask formed remains on top of each GaN micro-rod.

### X-ray diffraction rocking curve measurements

have been performed on all the samples as a function of azimuth angle in order to evaluate the crystal quality of non-polar GaN. The azimuth angle is defined as 0° when the projection of an incident x-ray beam on a nonpolar (11–20) GaN surface is parallel to the *c* direction of (11–20) GaN, while it is defined as 90° when the projection of the incident X-ray beam is along the [1–100] direction, i.e., *m* direction.
